# Cardio-Respiratory Coordination Increases during Sleep Apnea

**DOI:** 10.1371/journal.pone.0093866

**Published:** 2014-04-09

**Authors:** Maik Riedl, Andreas Müller, Jan F. Kraemer, Thomas Penzel, Juergen Kurths, Niels Wessel

**Affiliations:** 1 Cardiovascular Physics, Department of Physics, Humboldt-Universität zu Berlin, Berlin, Germany; 2 Department for Cardiology, Sleep Medicine Centre, Charité Universitätsmedizin Berlin, Berlin, Germany; 3 Research Domain on Transdisciplinary Concepts and Methods, Potsdam Institute for Climate Impact Research, Potsdam, Germany; Universitat Pompeu Fabra, Spain

## Abstract

Cardiovascular diseases are the main source of morbidity and mortality in the United States with costs of more than $170 billion. Repetitive respiratory disorders during sleep are assumed to be a major cause of these diseases. Therefore, the understanding of the cardio-respiratory regulation during these events is of high public interest. One of the governing mechanisms is the mutual influence of the cardiac and respiratory oscillations on their respective onsets, the cardio-respiratory coordination (CRC). We analyze this mechanism based on nocturnal measurements of 27 males suffering from obstructive sleep apnea syndrome. Here we find, by using an advanced analysis technique, the coordigram, not only that the occurrence of CRC is significantly more frequent during respiratory sleep disturbances than in normal respiration (p-value<10^−51^) but also more frequent after these events (p-value<10^−15^). Especially, the latter finding contradicts the common assumption that spontaneous CRC can only be observed in epochs of relaxed conditions, while our newly discovered epochs of CRC after disturbances are characterized by high autonomic stress. Our findings on the connection between CRC and the appearance of sleep-disordered events require a substantial extension of the current understanding of obstructive sleep apneas and hypopneas.

## Introduction

Frequent apneic and hypopneic events (AHE) during the night increase the risk of metabolic disorders, e.g. diabetes type 2, and cardiovascular diseases like hypertension and stroke [1]. As cardiovascular diseases are the main source of morbidity and mortality in the United States with costs of about $172 billion in 2010 [2], understanding the pathological regulatory mechanisms in AHE is of high priority for public health.

AHE are defined by a reduced or disrupted ventilation which is either caused by obstructions of the upper airways (obstructive event) or a cessation of the respiratory motor output from the brain stem (central event) for more than ten seconds [3], [4]. They are accompanied by oxygen desaturation and carbon dioxide accumulation in the blood and autonomic stress at their ends which is reflected by increasing heart rate, blood pressure and respiratory effort. The role of these effects in the pathogenesis of the co-morbidities is not fully understood yet, especially those of the autonomous activation which prevents recuperative sleep.

A decreased interrelationship of heart rate and respiration is used as an indicator of autonomic excitation. The most prominent feature of this interrelationship is a modulation called respiratory sinus-arrhythmia, i.e. the respective increase and decrease of the heart rate during inspiration and expiration [5], [6]. But there is also a mutual influence of the cardiac and respiratory oscillations on their respective onsets, in particular, leading to spontaneous cardio-respiratory coordination (CRC), a tendency towards a constant-time relationship between both onsets. Attention should be paid to the strict distinction between CRC and the respiratory sinus-arrhythmia. Because the first one is a triggering phenomenon in both directions and the second one results from a modulation of autonomic activity by respiration, traditional methods for the investigation of respiratory sinus-arrhythmia, such as the frequency analysis, are unable to quantify CRC. Cardio-ventilatory coupling [7]–[11], the alignment of the inspiratory onset with the heart beat, and phase synchronisation [7], [12]–[16], i.e. the adjustment of heart beats at phases of the respiratory cycles, are partial descriptions of this phenomenon and are mostly observed during anaesthesia [8], rest [11], [16], sleep [7], [10], [11], [13], [14] and controlled breathing [17]. As an example of disturbing stress, AHE [10], [14] as well as mental stress [18] are assumed to strongly reduce CRC.

**Figure 1:**
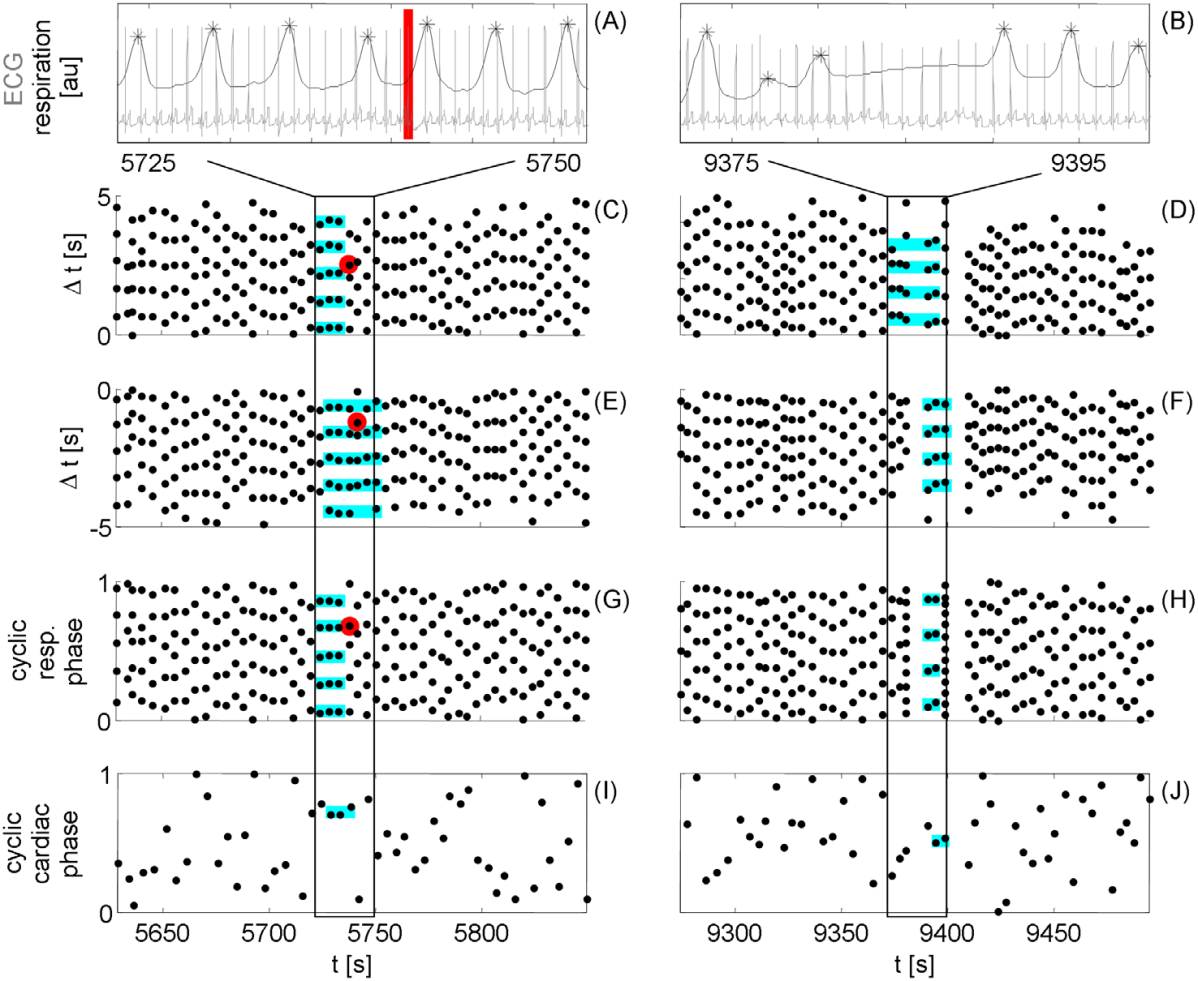
Examples of detected cardio-respiratory coordination in the presences of cardio-respiratory disturbances. The disturbances are an ectopic beat (left column - colour coded red) and a central apnea (right column). Panels A and B show epochs of the considered respiratory curve (black line) with the selected onsets (asterisks) and the electrocardiogram with the characteristic R-peak (grey). The columns of points in the plots below indicate the occurrence of these R-peaks: in the cycle after each respiratory onset set at 0s (C and D, IR-plot); in the cycle before each respiratory onset set at 0s (E and F, RI-plot); and in relation with the respiratory cycles after each respiratory onset characterized by the interval (G and H, synchrogram). In panel I and J the points represent the occurrence of the respiratory onset in relation to the including cardiac cycles. Blue stripes highlight parallel horizontal structures indicating cardio-respiratory coordination. The framed epoch in panel E shows an example of fluctuation of the number of heart beats per breath where the number of points in the columns varies between 4 and 5.

However, the origin of CRC is still under discussion. The analysis of disturbances of the cardio-respiratory system and of their effects on CRC could help to answer this question. Examples of such events are ectopic beats or the aforementioned AHE are shown in Fig. 1. In this picture, the four most prominent visualization tools for CRC, parallel horizontal lines indicate CRC, are shown (from top to the bottom): the IR-plot indicating the alignment of the cardiac onsets with the respiratory onset [7]–[9], [19], [20]; the RI-plot indicating the alignment of the respiratory onset with the cardiac cycles [8], [9], [19], [20]; the traditional synchrogam indicating cardiorespiratory phase synchronisation [7], [9], [12], [14], [16]; and the synchrogram used by Raschke et al. [10], [11]. A comparison of the RI- and the IR plot displays a higher ordering in the region of ectopic beats and central apnea, respectively, indicating different directions of coupling between heart rhythm and respiration. Both synchrograms hardly shows such ordering because of the rapid change of the length of the expiratory pause which influences the calculation of the respiratory phase. This problem is also reflected by the changing ratio of heart beats to respiratory cycles. Especially the increase of ordering for the central apnea is surprising since a reduction of CRC is assumed in this case [10].

Therefore, the occurrence of CRC during AHE is analysed in this study in order to evaluate the importance of CRC for AHE. To overcome the limitations of the aforementioned standard tools, we combine RI- and IR-plot to the so-called coordigram, considering both directions of influence, from heart beat to respiration and vice versa, simultaneously. Additionally, we change the quantification of parallel lines from a histogram, normally used to quantify the order in time plots, to a kernel estimated distribution allowing for a more time resolved consideration which is necessary to account for short sequences of CRC. One reason to expect short sequences is a high noise level that results from the errors in onset detection and the manual determination of the AHE. Therefore, we need a robust means to analyse the detected CRC events statistically.

## Methods

### Data and Pre-processing

We analyze nocturnal measurements of 27 males suffering from obstructive sleep apnea syndrome. The study was approved by the local ethics committee of the Charité in Berlin. Participants provided their written informed consent to participate in this study and the informed consent of all subjects was recorded in paper form. A total of 10814 AHE, almost all obstructive ones, are annotated according to the AASM manual [3], [4] by trained technicians on the basis of nasal airflow measure. The sleep stages are scored according to the recommendations of the AASM manual [4] and pooled to: awake - W, light sleep - LS, deep sleep - DS, and rapid eye movement sleep - REM. From the electrocardiogram (sampling rate 2000 Hz) the peak of the QRS complexes define the time of the cardiac onsets t_Ci_ (i = 1,…,N_C_ the number of heart beats; in Fig. 1 A,B for example), whereas the time of the respiratory onsets t_Rj_ (j = 1,…,N_R_ the number of respiratory cycles) are defined via the local maxima of the abdominal movements (sampling rate 10 Hz) as measured by respiratory belts (Fig. 1 A,B). The time points of the respiratory and the cardiac onsets as well as the status of each respiratory onset (present sleep stage; AHE yes/no) are provided by S1.zip. The abdominal signal is used instead of recommended airflow, since abdominal movement represents respiratory motor output even during full upper airway obstruction. We group the onsets into three categories: during AHE (in AHE), in intervals from one to a specific number of onsets after each AHE (after AHE) and normal onsets.

### Detection of Cardiorespiratory Coordination

**Figure 2:**
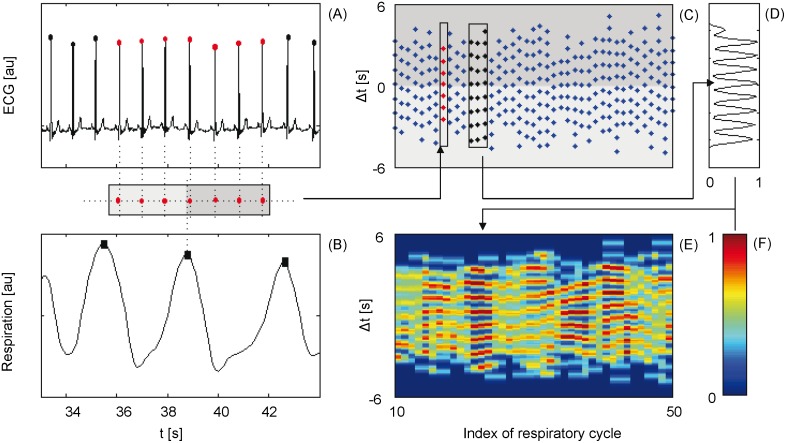
Scheme of the cardio-respiratory coordination detection. (A) Onsets of the cardiac cycles in the electrocardiogram (points). (B) Onsets of the ventilation in the respiratory signal (squares). (C) Each column of the coordigram reflects the temporal relation between a respiratory onset (zero) and the cardiac onsets in the two neighboured respiratory cycles. (D) From (C) an estimation of the point distribution in a sliding window over three respiratory events is estimated by means of the Equ. 1 and 2. (E) the amplitude of each distribution is colour coded by (F) which highlights the coordination patterns in C by red stripes (positive range of Δt: the heart triggers respiration; negative range of Δt: ventilation coordinates the heart).

In order to detect CRC, we propose a new analysis tool, the coordigram, which is a substantial extension of standard methods [7], [8] because it diminishes boundary effects and allows a comparison of the direction of interactions. The coordigram is generated by columns of points reflecting the temporal distance between a respiratory onset and the cardiac onsets in the preceding and following respiratory cycle (Fig. 2C). CRC is indicated by parallel horizontal dotted lines. Lines only in the negative range of Δt reflect the cardiac influence on the respiratory onset, whereas lines only in the positive range show respiratory control of the next cardiac onset. These parallel horizontal lines are quantified by the point distribution in sliding.

windows (Fig. 2D for example). The point distribution of the i-th respiratory onset is given by
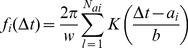
(1)This kernel estimation is based on the values of Δt in the sliding window around the i-th respiratory onset a_i_ = {Δt_k_}_k = i-w,…,i+w_ where Δt_i_ = {t_Ci_-t_Rj_}_j = 1,…,NC_. The chosen window length w of three respiratory cycles reflects the expectation that the status of the CRC changes rapidly, especially at the end of the AHE. The Gaussian kernel.
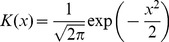
(2)is used with a bandwidth b = 0.2s, twice the sampling time of the respiratory signal, reflecting the assumed detection error of the respiratory onsets) [21]. The distribution is normalized in order to achieve a maximum value of the curve of 1, if the points lie on exact lines. CRC is assumed if the power of the spectral component of this estimation’s main oscillation is beyond a threshold ε, here ε = 0.0828, in the positive and/or the negative range of Δt. This is visually reflected in regular changes of red and blue stripes in the colour coded temporal evolution of the distribution (Fig. 2 E,F).

For the ease of comparison, the well-known synchrogram [16] is additionally calculated in our study. To create the synchrogam, the positive values Δt are normalized by the length of the corresponding respiratory cycle which conforms to the event based determination of the phase [16]. It reflects the synchronisation ratios n:m with m = 1, the number of breaths in the considered forcing cycle. A restriction which results from a test proposed by Porta et al. 2004 [22]. In the synchrograms, the structures of parallel horizontal lines are quantified as in the coordigram. Because of the normalization, the bandwidth of the Gaussian kernel has to be divided by the mean length of the breathing cycles, which is about 4 seconds. This results in b = 0.05 corresponding to the bin size of 0.03 in the Toledo method for detecting phase synchronisation [23].

### Statistics

CRC and phase synchronization are quantified by the number of respiratory onsets where the respective phenomenon is detected. Of course the amount of detected instances depends on the choice of the algorithm intrinsic parameters w, b, and ε. So, a decrease of w or ε leads to an increase of the detection rate of CRC. In the case of b, initially an increase of the value also increases the detection rate. But for higher values (b>0.2) this rate dramatically declines because of the increasing ratio of this value to the length of the heart beat intervals. However, a small value of w, as used in this analysis, increases detections of CRC by chance. Therefore we only compare relative frequencies of detection. Assuming a homogenous distribution of miss-detection, the number of detected instances in specific classes, i.e., sleep stage (W, LS, DS, REM) and/or respiratory status (normal, in AHE or after AHE), would have to be proportional to the percentage of occurrence of each class over the whole measurement. We compare the actual detected amount with the expected one using 2×2 contingency tables, where the class of normal respiratory status acts as a reference. That means, the number of respiratory onsets are counted for all possible combinations of characterized features: coordination (no CRC, CRC), respiratory status, and sleep stage. The 2×2 contingency tables are constructed by aggregating the numbers of each of the coordinated and uncoordinated onsets for a specific combination of respiratory status and sleep stage, and contrasting them with the corresponding numbers of onsets for normal respiratory status in the same sleep stage. Contingency χ2 tests are used to decide if there is an interrelation between the selected class and the occurrence of coordination (significance level = 0.05). Additionally, each 2×2 contingency table provides information about the influence of the selected feature combination on the occurrence of detected CRC. Because of the number of comparisons and the resulting multiple testing problem, the p-values are adjusted by the Bonferroni correction. We also performed a sensitivity analysis of the test where the influence of the detection parameters w, b and ε on the decision is considered.

## Results

**Figure 3:**
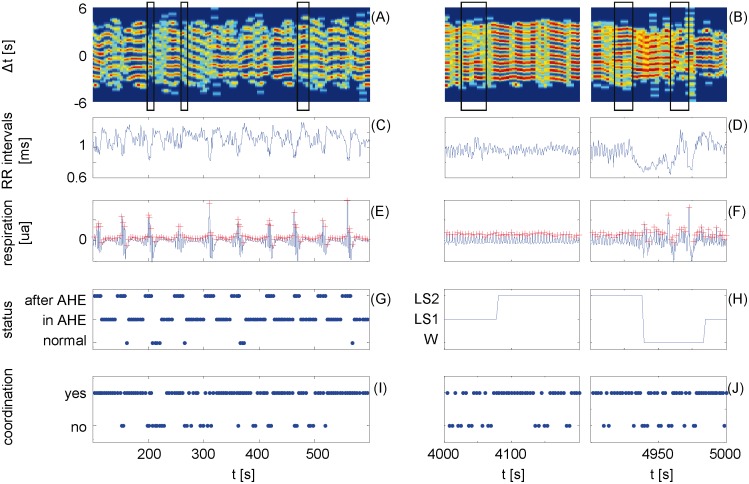
Examples of cardio-respiratory coordination during repetitive Apnea-Hypopnea-Events (AHE) and changes of sleep stages. For the AHE (left column) and changes of sleep stages (right column), the coordigram (A and B), the corresponding cardiac beat-to-beat intervals (C and D), the corresponding respiratory signal with the selected onsets (E and F), and marker of detected coordination (I and J) are plotted. Panel G displays the classification of the respiratory cycles in relation to respiratory events. Panel H shows the scored sleep stages where the light sleep LS was divided in S1 and S2 according to the AASM manual [14]. The frames in the panels A and B mark asymmetric structures which indicate unidirectional influences.

**Figure 4:**
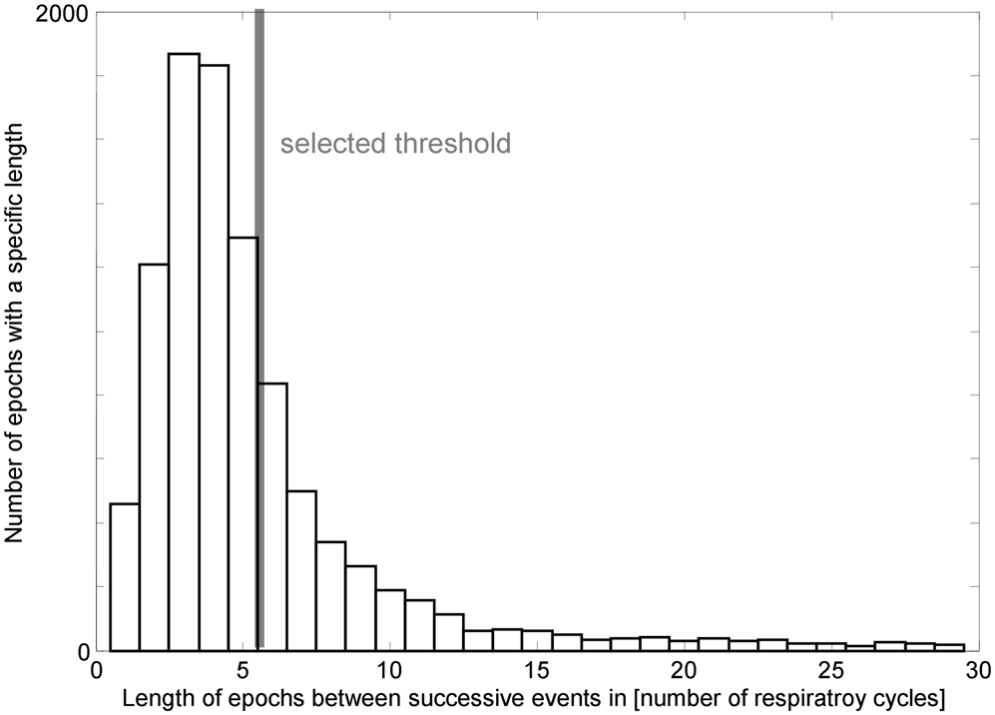
Distribution of the duration between successive Apnea-Hypopnea-Events. The duration is quantified by the number of respiratory cycles. The grey bar marks the selected threshold value bounding the periods after AHE.

The respiratory and cardiac onsets are extracted from the electrocardiogram and the simultaneously recorded signal of the abdominal movement, respectively. On that basis the new coordigram plot is built (see Fig. 2) indicating CRC in about 51% of the breathing cycles. It not only exhibits a mutual coordination of cardiac and respiratory onsets, but also one-way influences (e.g. Fig. 3 A and B). We divide the respiratory onsets into two groups: with and without CRC. Additionally, they are also classified according to the current respiratory state (normal, in AHE, and after AHE). The maximum number of respiratory onsets after AHE was set to five because of the dominantly short epochs between successive AHE (see Fig. 4). Further, the respiratory onsets are divided into groups in respect of the sleep stage (awake - W, light sleep - LS, deep sleep - DS, and rapid eye movement - REM) where they occurred. Examples of the three different classifications are illustrated in Fig. 3. Table 1 shows the total number of respiratory onsets in each of the combined classes. The frequencies depend on the algorithmic parameters. To increase the independence of these parameters, we test the proportion of CRC for the different groups of respiratory onsets in relation to normal respiration by means of 2×2 contingency tables. The null hypothesis of the following tests refers to a random distribution of the detected CRC, whereas the alternative implies a significant increase or decrease (see Table 1). The tests show a higher number of occurrences of detected CRC when compared to normal breaths:

- during AHE (p<10^−51^), and,
- after AHE (p<10^−15^).

**Table 1:** Quantitative evidence of the differences between cardio-respiratory coordination (CRC) and phase synchronisation (PS).

respiration	light sleep			deep sleep			rem		
	noCRC	CRC	PS	noCRC	CRC	PS	noCRC	CRC	PS
Normal	22227	23741	1653	9286	11896	1512	7284	7630	343
in AHE	15539	20604^†^	651*	2544	2995^†^	134*	2942	3833^†^	116*
after AHE	14112	15818^†^	670*	2067	2063*	115*	1971	2327^†^	64*

The table shows the total number of respiratory cycles in the considered situations of respiration and sleep stages. The symbols † and * indicate significant proportional increases and decreases, respectively, in and after apneic and hypopneic events (AHE) in comparison to normal breaths (2×2 contingency tables, χ^2^-test, significance level α = 0.05 with Bonferroni correction.).

(A value of 10^−51^ is the lowest computable significance level of our statistical tool.) illustrated in Fig. 3. Testing both group differentiated by means of the sleep stage leads to the results of an increased occurrence of CRC (indicated by the symbols †) shown in Table 1. Applying the quantification mechanism for phase synchronisation, we find, conversely, a significant decrease of occurrences (indicated by the symbol *, Table 1).

Furthermore, Fig. 3 gives an example of the asymmetric order of the coordigram in the presence of changing sleep stages which indicates unidirectional coordination of heart rhythm and respiration.

Variation of the detection parameters b, w, and ε lead to marginal changes in the testing outcome which are base on the changes of the detection rate. So, growing values of ε and w lead to decreasing rates. In the case of b, there is an increase of the rate until b = 0.15 after which the rates decrease. All these trends are faster during disturbed respiration than during normal breaths resulting in changes of the test’s outcome. The null hypothesis is not rejected in the case of respiratory cycles after AHE during REM sleep if w>3 or ε≥0.09. The same holds for respiratory cycles in and after AHE during deep sleep for ε≤0.07; in AHE during REM for w≥11; and after AHE during LS for ε = 0.09. In the last case, ε = 0.09 marks a change of the statistical outcome. Lower values lead to a significantly higher detection rate while larger ones lead to a significantly lower detection rate in the respiratory onsets after AHE during LS in comparison to during normal breaths in the same sleep stage.

Using our innovative combination of standard time domain plots, and a time resolved analysis technique, we are able to show for the first time that the number of respiratory cycles indicating CRC during AHE is disproportionately large compare to normal respiration. Moreover, CRC is also significantly increased under forced autonomic stress after AHE (e.g. Fig. 3A) [24]. These findings of our proposed approach differ greatly from past ones that resulted from global considerations [10]. Therefore, CRC seems to be an important mechanism for the development of AHE. Finally, we demonstrate that the observed CRC is quite different from cardio-respiratory synchronisation which decreases during AHE.

## Discussion

In summary, cardio-respiratory coordination has to be considered as a phenomenon which not only appears in resting conditions but also under high autonomic stress at the end of AHE. This contradicts past findings [11] and clearly shows the advantage of the novel analytical tool, the coordigram. Raschke et al. 1986 [10] propose increasing metabolic transport as a main cause of diminished CRC during physical activity. Later, Bartsch et al. 2012 [13] suggest a sympathetic dominance of the excitatory branch as a reason for decreased cardiorespiratory synchronisation. Our results, however, show that CRC increases at the end of AHE, in spite of increased sympathetic activity and the metabolic transport. The underlying mechanism is unknown but it seems to be independent of the respiratory sinus-arrhythmia because the example in Fig. 3 shows a similar rate of CRC in cases with different levels of respiratory modulation in the beat-to-beat intervals. The most popular model of sequential apneas is based on an instability of the chemical feedback control of blood which leads to rhythmic changes of breath (e.g. central apnea) [25] or an uneven distribution of the respiratory neural output to chest wall and upper airway muscles (obstructive events) [26]. However, it remains unclear why the occurrence of AHE, especially serial ones, is concentrated in LS although there is a similar chemoreflex responsiveness as in DS. Obviously, the significant differences between both sleep stages in the occurrence of CRC after AHE implies a necessary condition for serial AHE. Further on, this importance is also indicated by the increased appearance of CRC in AHE. Therefore, we hypothesize that CRC is important for the development of AHE. This hypothesis could explain the observation of Wallois et al. 2008 [27] who describe the case of a neonate where CRC was observed before a life-threatening cessation of breath. Recent studies show the evidence of two generators of spontaneous breathing patterns in the brainstem, the so-called pre-Bötzinger complex (preBö) and parafacial respiratory group (pFRG) [28], [29]. The neurons of pFRG are associated with the expiratory rhythm generator “vetoing” the inspiratory bursts of preBö [29]. We hypothesize the importance of the pFRG for the dominant part of the detected CRC where the start of the inspiration aligns to the heart beats. This would require a connection of the cardiovascular control with pFRG, perhaps via the afferent baroreceptor activity [30]. But this still needs to be investigated. Furthermore, the preBö is assumed to be the main contributing factor of the cardiac alignment to respiration the other part of CRC. We observed preBö’s dominance after AHE which would explain the increasing prevalence of AHE in older people because of increasing importance of preBö for the respiratory rhythm during aging [28]. This dominance could be a reason for the fast overshoot of the vagal activity after the AHE resulting in a new event. In DS we see a drastic reduction in the number of AHE and a significant decrease in CRC after AHE (see Table 1). Similar to several studies which hypothesize that the activity of preBö is depressed by opiates [28], [29], it is possible that DS could have a similar depressing effect on preBö as this analgesic state.

The comparison of CRC and phase synchronisation illustrates the need of a more differentiated characterization of the temporal alignment of heart beats and breathing.

The main reason for reduced detection of phase synchronisation using traditional means are complex fast changes of the whole-numbered ratio of heart beats and breathing cycles described and classified by Galletly et al. 1997 [8]. This phenomenon indicates a need for a more extensive description of the self-organization in networked relaxation oscillators. These oscillators, e.g. respiratory neurons [28], [29] and the cardiac pacemaker cells, are characterized by fast temporary discharges and longer threshold-depending recovery times. As shown in computer models, relaxation oscillators are capable of a rapid coordination [31] which is also shown in our results, especially during and after AHE. In the analyzed example, this coordination seems to influence rhythmic changes of slower periods than the eigenfrequency of the coupled oscillators, e.g. series of AHE [26]. So, the fast stepwise change of the whole-numbered ratio of heart beats and breathing cycles is a cascade of period lengthenings in both oscillators. This clearly overshoots the range of both eigenfrequencies which then results in a recovery period.

The example of CRC in the presence of changing sleep stages (see Fig. 3) indicates two findings. First, AHE and ectopic beats are not the only disturbances which can be used to analyze the mechanisms behind the cardio-respiratory coordination. This emphasizes not only the sensitivity of CRC to disturbances but also its very fast recovery after these events.

Second, panels B and D elucidate the limitation of our quantification where stripes start overlapping. Here, the parameter b, the assumed detection error of the respiratory onset, is too large in relation to the length of the beat-to-beat intervals (∼600 ms). Only a more precise determination of the respiratory onset could counter this phenomenon and make the approach applicable for diurnal studies where beat-to-beat intervals of a length of about 600 ms are common. The sensitivity analysis confirms this trend, where higher values than b = 0.2 resulted in a strong reduction of detection. Furthermore, the sensitivity analysis uncovered the influence of the detection parameters on the testing results which are caused by the faster decay of the detection rate in disturbed respiration. An explanation could be a higher noise level in these cases. Sources of this additional amount of noise could be a higher body movement after AHE, and a worse detection of the respiratory onset in AHE in comparison with normal respiration. The higher noise level causes a stronger covering of the coordination and earlier rejection if the requirements for coordination become stricter. So, there is a trade-off between detecting all existing coordination events (small values, especially of ε) and avoiding missed detections (high values, especially of ε). The chosen parameter values represent a compromise.

However, the consideration of CRC may lead to innovations in various topics such as the obstructive sleep apnea syndrome [25], the sudden infant death syndrome [27], [28] as well as in the field of central respiratory arrest in sleep [28]. The proposed analysis tool, the coordigram, will definitely play a major role in these fields.

## Supporting Information

Data S1 **The time points of the respiratory and the cardiac onsets as well as the status of each respiratory onset (present sleep stage; AHE yes/no).**
(ZIP)
